# Anpassung der Infektionsschutzmaßnahmen im öffentlichen Dienst aufgrund der COVID-19-Pandemie: Handlungsempfehlungen für technische, organisatorische und persönliche Schutzmaßnahmen zur Reduktion der Infektionsausbreitung von SARS-CoV-2

**DOI:** 10.1007/s40664-020-00418-2

**Published:** 2021-01-07

**Authors:** Julia Pieter, Wibke Körner, Volker Harth, Alexandra M. Preisser

**Affiliations:** Zentralinstitut für Arbeitsmedizin und Maritime Medizin (ZfAM), Universitätsklinikum Hamburg-Eppendorf, Seewartenstr. 10, 20459 Hamburg, Deutschland

**Keywords:** Arbeitsschutz, STOP-Prinzip, Epidemie, Polizei, Feuerwehr, Occupational safety, STOP principle, Epidemic, Police, Fire brigade

## Abstract

Die COVID-19-Pandemie führt zu veränderten Anforderungen an den Arbeitsschutz am Arbeitsplatz. Ziel der bundesweit durchgeführten Maßnahmen ist es, das Risiko einer Verbreitung der Virusinfektion zu reduzieren. Dies gilt in allen Lebensbereichen, also auch am Arbeitsplatz. Der Tätigkeitsbereich „Öffentlicher Dienst“ umfasst viele systemrelevante Berufsgruppen. Hierzu zählen u. a. Polizei, Feuerwehr, Justiz, Stadtreinigung, Wasserwerke sowie Verwaltungsorgane auf Stadt- und Landesebenen. Es besteht eine große Diversität der Tätigkeiten in Kommunal- und Landesbetrieben, insbesondere in Bezug auf die innerbetriebliche Organisation sowie personelle und räumliche Gegebenheiten. Häufig sind Interaktionen mit der Bevölkerung notwendig. Die Aufrechterhaltung der Funktionsfähigkeit dieser Strukturen ist essenziell für das öffentliche Leben. Bildungsbetriebe und Betreuungseinrichtungen für Kinder, Menschen mit Behinderungen und vulnerable Gruppen, Einrichtungen der Kranken- und Altenpflege sowie der öffentliche Personennahverkehr werden aufgrund der anderen Arbeitsstrukturen in diesem Statement nicht behandelt. Diese Handlungsempfehlung richtet sich an die verantwortlichen Personen in den jeweiligen Institutionen des öffentlichen Dienstes. Sie befasst sich mit den Maßnahmen des Infektionsschutzes während der Arbeit im öffentlichen Dienst, abgeleitet aus den Gefährdungsbeurteilungen verschiedener Settings. Die vorgestellten Maßnahmen können im Rahmen eines betrieblichen Konzeptes für zeitlich befristete Maßnahmen zum Infektionsschutz umgesetzt werden. Ziel ist es, allgemeine Hinweise zum Infektionsschutz in Bezug auf Arbeitssituationen zu geben, um die Verbreitung von SARS-CoV‑2 zu verlangsamen, Risikogruppen zu schützen und die Funktionsfähigkeit der genannten Strukturen zu gewährleisten.

## Abstract (en)

The COVID-19 pandemic is changing the requirements for occupational health and safety in the workplace. The aim of the measures implemented nationwide is to reduce the spread of the virus. This applies to all areas of life, including the workplace. The “public service” includes many system-relevant occupational groups: police, fire brigade, judiciary, municipal cleaning, waterworks and administrative bodies at local and state levels. There is a broad diversity of activities in communal and federal state enterprises, especially with regard to internal organisation as well as personnel and spatial conditions. Direct interactions with people are often necessary. Maintaining the functionality of these structures is essential for public life. Education institutions and care facilities for children, people with handicaps and vulnerable groups, institutions for nursing and geriatric care as well as public transport are not covered in this statement due to their special work structures. These recommendations address the professionally accountable in the respective public service institutions. It deals with infection control measures at work in the public service, derived from the risk assessments of various settings. The measures presented can be implemented as part of an operational concept for temporary infection control measures. The aim is to provide general guidance on infection protection with respect to work situations in order to slow down the dissemination of severe acute respiratory syndrome coronavirus 2 (SARS-CoV-2), to protect at-risk groups, and ensure the functioning of the aforementioned structures.

In der Erklärung des Bundesministeriums für Arbeit und Soziales zu SARS-CoV-2-Arbeitsschutzstandards [2] wird die Erstellung eines betrieblichen Konzeptes für zeitlich befristete Maßnahmen zum Infektionsschutz verlangt. Hierfür muss die bestehende Gefährdungsbeurteilung [3] angepasst und erweitert werden. In einem betrieblichen Pandemieplan sollen Regelungen zu verschiedenen innerbetrieblichen Situationen festgelegt und Ansprechpartner und Verantwortliche benannt sein.

*Die SARS-CoV-2-Pandemie erfordert eine zeitlich befristete Erweiterung der betrieblichen Gefährdungsbeurteilung und Arbeitsschutzmaßnahmen.*

Diese Publikation hat den Zweck, die bereits lokal und regional bestehenden Handlungsanweisungen spezifischer zu gestalten. Sie soll dazu beitragen, die Tätigkeiten im öffentlichen Dienst für die Mitarbeitenden möglichst sicher sowie physisch und psychisch wenig belastend im Hinblick auf SARS-CoV-2-Infektionen zu gestalten. In einer ausführlicheren Version dieses Papiers auf der Homepage des Kompetenznetzes Public Health COVID-19 [4] finden sich ergänzende Checklisten für verschiedene Arbeitsbereiche im öffentlichen Dienst.

## Methoden und Lösungsansatz

Die Autor(inn)en haben eine webbasierte Suche nach aktuellen Empfehlungen und Erkenntnissen zu dem Thema und benachbarter Themen durchgeführt, diese zusammengeführt und durch eigene, erfahrungsgestützte Detailüberlegungen ergänzt. Als Lösungsansatz wurden die bereits veröffentlichten und verordneten Hygienemaßnahmen zur COVID-19-Pandemie mit Arbeitsschutzmaßnahmen entsprechend des STOP^1^-Prinzips kombiniert. Vorliegende Empfehlungen beruhen auf der Meinung von mehreren Expert*innen sowie Vorgaben der entsprechenden Behörden. Es gibt zum derzeitigen Zeitpunkt keine quantitativ ausreichende Evidenz für die Wirksamkeit der Schutzmaßnahmen im Kontext von Infektionen mit SARS-CoV-2. Die Darstellung entspricht dem Stand der Wissenschaft (Juni 2020).

## Gefährdung der systemrelevanten Bereiche des öffentlichen Dienstes durch Häufung von COVID-19-Infektionen

In Anlehnung an die Definition kritischer Infrastrukturen [1] sind verschiedene gesellschaftliche Bereiche, Organisationen oder Einrichtungen, deren Ausfall oder Beeinträchtigung nachhaltig wirkende Versorgungsengpässe, Störungen der öffentlichen Sicherheit oder andere dramatische Folgen nach sich ziehen würde, vom Bundesministerium für Arbeit und Soziales aufgelistet worden. Hierunter fallen u. a. die Energie- und Wasserversorgung, die Müllentsorgung, Transport und Verkehr, Schulen, Kinder- und Jugendhilfe, das Finanzwesen, die Gesundheitsversorgung und die staatliche Verwaltung auf Bundes‑, Landes- und Kommunalebene [5].

*Systemrelevant sind alle Bereiche, die eine Schlüsselrolle für die Stabilität der Gesellschaft, der Wirtschaft und des staatlichen Gemeinwesens haben.*

In dieser Publikation werden hauptsächlich die Bereiche des öffentlichen Dienstes behandelt, welche sich der staatlichen Verwaltung zuordnen lassen, nämlich Polizei, Feuerwehr, Stadtreinigung sowie Verwaltungsorgane auf Kommunal- und Landesebenen. Nicht behandelt werden – aufgrund der besonderen Anforderungen und anderen Arbeitsstrukturen – Bildungsbetriebe, Betreuungseinrichtungen für Kinder, Menschen mit Behinderungen und vulnerable Gruppen sowie der öffentliche Personennahverkehr. Insbesondere für Tätigkeiten in Einrichtungen der Krankenversorgung und Altenpflege bestehen, durch den möglichen Kontakt mit SARS-CoV-2-Infizierten und regelmäßigem engen Kontakt zu Angehörigen von Risikogruppen für einen schweren Verlauf von COVID-19, spezielle Anforderungen an den Infektionsschutz; auch diese werden in dieser Handlungsempfehlung nicht behandelt.

### Sicherstellung der Funktionsfähigkeit von systemrelevanten Bereichen durch Regelungen zum Personalmanagement

Die Beschäftigten aus systemrelevanten Berufsgruppen haben nichtvermeidbare berufsbedingte Kontakte zu Kolleg*innen sowie zur Bevölkerung, woraus ein erhöhtes Infektionsrisiko resultiert. Personalausfälle aufgrund von Quarantänemaßnahmen im Rahmen des nationalen Pandemiemanagements erschweren die Aufrechterhaltung des normalen Betriebs [6]. Unternehmen der kritischen Infrastruktur müssen im betrieblichen Pandemieplan unter den Beschäftigten Schlüsselfunktionen (nichtverzichtbare Funktionen) identifizieren und deren Verfügbarkeit durch Stellvertretungsregelungen sicherstellen. Der physische Kontakt des vom Unternehmen zu identifizierenden Schlüsselpersonals zum Rest der Belegschaft sollte – soweit möglich – eingeschränkt werden [6]. Das Robert Koch-Institut (RKI) regelt eine Ausnahme für den Fall von Personalmangel in einzelnen systemrelevanten Bereichen im Rahmen der aktuellen Pandemie: Zur Sicherstellung der Funktionsfähigkeit kann im Quarantänefall (Kontaktpersonenmanagement oder bei akuten Erkältungssymptomen) Schlüsselpersonal unter Auflagen eingesetzt werden (Selbstbeobachtung, Mund-Nasen-Schutz [MNS] während der gesamten Anwesenheit am Arbeitsplatz, Hygienemaßgaben wie häufiges Händewaschen, sofern die Tätigkeit dies nicht zwingend ausschließt, Abstand zu anderen Personen (mind. 1,5 m) halten, Testung auf SARS-CoV‑2, wenn Symptome passend zu den RKI-Kriterien vorliegen; [7, 8]).

Betriebliches Pandemiemanagement bedarf expliziter interner Ansprechpartner*innen und geregelter, klarer Kommunikation.

Die Kommunikation der Arbeitgeber*innen bezüglich der betrieblichen Maßnahmen soll zielgruppen-spezifisch gut verständlich sein (betriebsinterne Medien, E‑Mail-Verteiler, Dienstanweisungen, Unterweisungen, Plakat-Aktionen, Aushänge, Schulungen u. a.). Wichtige Ansprechpartner*innen sollen benannt und die Kontaktdaten allen Mitarbeitenden zugänglich sein. Bei der Festlegung und Umsetzung der Maßnahmen sollen auch die Arbeitnehmervertretung hinzugezogen sowie Sorgen und Fragen der Beschäftigten berücksichtigt werden [9]. Die Gefährdungsbeurteilung muss auch die geänderten Anforderungen und die sich daraus ergebende psychische Belastung der Beschäftigten berücksichtigen [10–12].

Zusätzlich kann eine arbeitsmedizinische Beratung durch den/die Betriebsmediziner*in zum individuellen Risiko der Beschäftigten für einen schweren Verlauf einer COVID-19-Erkrankung auch telefonisch angeboten werden. Die Zugehörigkeit zu einer Risikogruppe kann dazu führen, dass eine Umsetzung an einen Arbeitsplatz mit geringerem Risiko für eine Ansteckung (Home Office) oder auch eine (befristete) Freistellung von der beruflichen Tätigkeit erforderlich wird, wenn sich das betriebliche Infektionsrisiko nicht senken lässt. Weitere Informationen zu Risikogruppen finden sich auf der Homepage des RKI [13] und auch in den Stellungnahmen „Beschäftigte mit erhöhtem Krankheitsrisiko“ [14] und „Müssen ältere Beschäftigte dem Arbeitsplatz fernbleiben?“ des Kompetenznetz Public Health COVID-19 [15].

Screeninguntersuchungen, wie tägliche Temperaturmessungen bei Beschäftigten, sind nicht geeignet, um Infizierte zu erkennen und eine Ausbreitung von SARS-CoV‑2 aufzuhalten [16]. Umso wichtiger sind eine individuelle Gefährdungsbeurteilung und Etablierung geeigneter Arbeitsschutzmaßnahmen.

## Arbeitsschutzmaßnahmen zur Risikominderung einer Infektion mit SARS-COV-2 in verschiedenen Arbeitsszenarien des öffentlichen Dienstes

Ein typischer Aspekt der Arbeit von Polizei, Feuerwehr, Stadtreinigung und Verwaltung ist der direkte Kontakt zu anderen Personen in verschiedener Intensität (bzgl. körperlichem Abstand und Kontaktzeit, kontrollierbare und weniger kontrollierbare Kontakte) und räumlich enge Arbeit in Teams. Dazu gehören:

- Beschäftigte mit direktem Kundenkontakt, deren Verhalten kontrolliert und kontrollierbar ist (z. B. in der Verwaltung),
- Beschäftigte mit direktem Kontakt zu Personen, deren Verhalten teils unkontrolliert oder unkontrollierbar ist (z. B. Polizei),
- Beschäftigte, die in Teams arbeiten (z. B. Mannschaft eines Löschzuges),
- Beschäftigte, die zusammen Verkehrsmittel benutzen (z. B. Müllfahrzeug).

Erster Schritt zur Festlegung von Arbeitsschutzmaßnahmen stellt die gesetzlich verankerte Gefährdungsbeurteilung des Arbeitsplatzes dar [3]. Hierin werden besondere Gefährdungsszenarien festgehalten und diese mit dem substanzspezifischen Risiko – hier der Infektion mit dem Virus SARS-CoV‑2 [17] – abgeglichen. Aus der Gefährdungsbeurteilung werden Maßnahmen des Arbeitsschutzes entwickelt, hier also des Schutzes vor der SARS-CoV-2-Infektion entsprechend der Biostoffverordnung. Im Arbeitsschutz wird das o. g. *STOP-Prinzip* angewendet, welches die Hierarchie der Schutzmaßnahmen festlegt und diese gruppiert. Diese Hierarchie der Schutzmaßnahmen ist auch bei den notwendigen Infektionsschutzmaßnahmen anzuwenden: An erster Stelle steht die vollständige Vermeidung der Gefährdung (Substitution); im Folgenden sind weitere Präventionsmaßnahmen der Verhältnisse anzustreben (technische und organisatorische Maßnahmen). Kann durch diese Maßnahmen der Verhältnisprävention kein ausreichender Schutz gewährleistet werden, müssen zusätzliche persönlich anzuwendende Schutzmaßnamen ergriffen werden (persönliche Schutzmaßnahmen). Die letztgenannten Maßnahmen der Verhaltensprävention stellen eine größere Beeinträchtigung der Mitarbeitenden dar und erfordern zudem die persönliche Motivation und Umsetzung jedes/jeder Einzelnen.

## Maßnahmen des Infektionsschutzes im öffentlichen Dienst

Viele der im Weiteren aufgelisteten Vorgehensweisen sind Maßnahmen des allgemeinen bevölkerungsbezogenen Infektionsschutzes, welche als temporäre Konzepte während der SARS-CoV-2-Pandemie auch im betrieblichen Kontext konsequent angewendet werden sollten.

### Generelle Grundsätze des Infektionsschutzes vor SARS-CoV-2-Infektionen

1. Kontakt reduzieren.
2. Abstandsregeln einhalten im Arbeitsteam und im Kundenkontakt.
3. Hygieneregeln beachten, u. a. regelmäßiges Händewaschen, Hustenetikette und kontaktfreie Begrüßungen.
4. Reduzierung eventueller Miterkrankungen oder Quarantänefälle durch Einrichten von festen Teams.
5. Persönliche Schutzmaßnahmen ergreifen, wenn erforderlich.

Diese Grundsätze können im Hinblick auf das STOP-Prinzip wie im Folgenden beschrieben umgesetzt werden.

### Substitution

Am effizientesten kann die Reduzierung möglicher infektiöser Kontakte mittels Substitution erreicht werden. Hierzu müssen bisherige Arbeitsweisen überprüft und nach Möglichkeit durch andere Prinzipien ersetzt werden: z. B. ein vermehrtes Anbieten telefonischer Beratungen anstelle eines persönlichen Kontaktes vor Ort. Schulungen, Teamsitzungen und Besprechungen können per Video- oder Telefonkonferenz ausgerichtet werden. Bürger*innen wird ermöglicht, Anträge online statt persönlich im Kundenzentrum zu stellen. Auswärtige Meetings können durch Videokonferenzen ersetzt werden, wodurch auch zusätzliche Reisekontakte vermieden werden.

### Technische Anpassungen

Technische Anpassungen betreffen die räumlichen und technischen Gegebenheiten für die Durchführung verschiedener Infektionsschutzmaßnahmen. Die Durchführung von Home Office erfordert die Einrichtung der technischen Voraussetzungen. Bei einer Tätigkeit im Büro oder einer vergleichbaren Arbeitsstelle sollten personenbezogene Arbeitsplätze festgelegt werden und die Benutzung von Tastatur, Maus etc. durch mehrere Personen vermieden werden.

Die Anpassung der räumlichen Gegebenheiten kann z. B. das Einführen von Trennwänden, Abstandshaltern und Plexiglastrennscheiben bei Kundenkontakt sowie den Zugang zu Händewasch-Möglichkeiten mit ausreichend Seife und Papier beinhalten. Um Kontaktflächen wie Türklinken zu reduzieren, sollten Durchgangstüren soweit möglich automatisch öffnen oder offengelassen werden.

Reinigungspläne [18] sind anzupassen; z.B. mit regelmäßiger Reinigung bestimmter Kontaktflächen. Die Reduktion der Handkontamination durch eine konsequente Umsetzung der Händehygiene ist die wirksamste Maßnahme gegen die Übertragung von Krankheitserregern auf oder durch Oberflächen [18]. Wenn ein Händewaschen nach Kundenkontakt nicht möglich ist, sollte alternativ Desinfektionsmittel in transportabler Flaschengröße bereitgestellt werden.

Eine regelmäßige und gute Belüftung der Arbeitsräume soll die Aerosolbelastung in der Innenraumluft reduzieren [2]. Auch durch den Betrieb von Raumluftanlagen kommt es zu einer Reduktion der Virenlast in einem Raum. Die Erhöhung des Außenluftanteils in Raumluftanlagen wird empfohlen. Eine Übertragung von Corona-Viren über Lüftungs‑/Klimaanlagen kann nach aktuellem Kenntnisstand ausgeschlossen werden [19].

### Organisatorische Anpassungen

Die Umsetzung der Infektionsschutzmaßnahmen erfolgt über organisatorische Anpassungen: Unterweisung der Mitarbeitenden über die betrieblichen Maßnahmen zum Infektionsschutz, die richtige Verwendung von Schutzkleidung, falls erforderlich, und das Verhalten im Krankheitsfall. Auch die Überprüfung der Schichtmodelle und die Strukturierung der Schichtwechsel zur Reduktion der Kontaktzeiten zwischen Mitarbeitenden bei Schichtwechsel sind erforderlich.

In Großraumbüros sollte die Anzahl der anwesenden Personen reduziert werden. Pro Person sollten ca. 9 qm Fläche in einem Raum vorhanden sein (Abstand von 1,5 m in alle Richtungen: 3 × 3 m). Mögliche organisatorische Anpassungen zur Reduktion der Personenanzahl pro Raum sind die weitestgehende Gewährung von Home Office sowie das wechselseitige Nutzen gemeinsamer Büroräume durch wochenweise Planungen von Home Office und Anwesenheit. Eine zeitliche Entzerrung der Einsätze der Mitarbeitenden kann durch versetzte Arbeitszeiten (Kernarbeitszeiten werden aufgehoben) oder Einführung von Schichtarbeit ermöglicht werden.

Die Einteilung fester Teams (hierbei auch Zuordnung von Auszubildenden oder Praktikant*innen zu festen Arbeitspartner*innen) ist eine sinnvolle Maßnahme, um im Erkrankungsfall die Zahl der Kontaktpersonen begrenzt zu halten. Eventuelle Infektionsermittlungen können hierdurch effektiver durchgeführt werden. Hierzu gehört auch eine konsequente räumliche und personelle Trennung kooperierender Arbeitsbereiche, so sollten z. B. Feuerwehr-Mitarbeitende, die im Rettungsdienst eingesetzt werden, möglichst räumlich von den Feuerwehr-Mitarbeitenden im Löschdienst getrennt werden. Die räumliche Trennung der einzelnen Teams sollte auch während der Pausenzeiten eingehalten werden.

Das Vorgehen bei Auftreten eines COVID-19-Erkrankungsfalls im Team sollte festgelegt und kommuniziert werden. Feste Ansprechpartner*innen für Hygienefragen und für den Fall einer Erkrankung im Team sollten benannt werden. Ebenso sollten die privaten Telefonnummern der Teammitglieder erfasst werden, damit zu ihnen zügig Kontakt aufgenommen werden kann.

### Persönliche Schutzmaßnahmen und Verhaltensregeln

Persönliche Schutzmaßnahmen und Verhaltensregeln erfordern die Mitarbeit jedes/jeder Einzelnen. Hierzu zählen die allgemeinen Verhaltensregeln des Infektionsschutzes, welche durch das Robert-Koch-Institut [20, 21] und die Bundeszentrale für gesundheitliche Aufklärung [22] veröffentlicht werden.

Die Mitarbeitenden sollen den direkten Kontakt zu anderen Personen meiden. Auf die Einhaltung eines Abstandes zu anderen Personen von mindestens 1,5 m sollen die Mitarbeitenden im Kontakt zu Kund*innen, innerhalb des Teams und auch während der Pausenzeiten achten. Die oben beschriebenen Hygieneregeln sollen befolgt werden (Händewaschen, Husten- und Niesetikette). Mitarbeitende mit Symptomen einer akuten Atemwegserkrankung sollen dem Dienst fernbleiben.

Dienstkleidung sollte in den Bereichen mit häufigen Personenkontakten regelmäßig gereinigt und gewechselt werden. Das Tragen einer Mund-Nasen-Bedeckung (MNB) und eines medizinischen Mund-Nasen-Schutzes (MNS) dient dem Fremdschutz, da das Verbreiten von Tröpfchen beim Sprechen und Husten abgeschwächt wird und somit das Infektionsrisiko für die Kontaktperson sinkt. Die Unterschiede zwischen MNS, FFP2-/FFP3-Masken und MNB werden im epidemiologischen Bulletin 19/2020 des RKI [20] und in der Stellungnahme der Deutschen Gesellschaft für Pneumologie und Beatmungsmedizin (DGP) zur „Auswirkung von Nase-Mund-Masken auf den Eigen- und Fremdschutz bei aerogen übertragbaren Infektionen in der Bevölkerung“ [23] erläutert und hier kurz zusammengefasst. Bei einer Mund-Nasen-Bedeckung (MNB) handelt es sich um eine möglichst doppellagige Stoffmaske. Die Durchlässigkeit bei MNB ist nicht normiert, da hier verschiedene Stoffarten zum Einsatz kommen. Einzelne Gewebe erreichen eine ähnliche Filterwirkung wie ein medizinischer MNS, insbesondere bei dicht gewebten Materialien. Der Atemwiderstand für den Maskenträger steigt bei sehr dicht gewebten Stoffen an. Die Schutzwirkung nimmt hingegen bei Durchfeuchtung des Materials ab. Ein medizinischer MNS bietet im Vergleich zu einer MNB eine geprüfte Durchlässigkeit und muss Mindestanforderungen erfüllen. Ein weiterer Vorteil eines medizinischen MNS ist, dass der Atemwiderstand geringer ist als dichtgewebte MNB mit ähnlicher Durchlässigkeit. Das Material eines MNS ist beschichtet, um eine Durchfeuchtung zu verlangsamen. Im beruflichen Kontext ist aus Sicht der Autor*innen daher ein MNS vorzuziehen, wenn verfügbar.

Für spezielle berufliche Einsätze mit erhöhter Infektionsgefährdung sollte die persönliche Schutzausrüstung einen Eigenschutz der/des Beschäftigten gewährleisten. Für den Eigenschutz ist das Tragen von FFP(„filtering face piece“)-Masken erforderlich. Auch hierbei handelt es sich um Halbmasken mit standardisierten Filtereigenschaften. Zum Schutz vor einer Erkrankung bei luftübertragbaren Erregern soll mindestens eine FFP2-Maske getragen werden [24]. Noch kleinere Partikel werden durch FFP3-Masken aufgehalten, daher ist diese in Einzelfällen bei sehr engem Kontakt zu einer infizierten Person erforderlich. Bei FFP2- und FFP3-Masken ist zu beachten, dass auch der Atemwiderstand steigt; Empfehlungen zur maximalen Tragedauer und einzuhaltenden Pausenzeiten sind zu beachten [25, 26]. Eine FFP2-Maske mit Ausatemventil schützt den Maskenträger, jedoch nicht die Kontaktpersonen, da der Ausatemstrom durch das Ventil nicht gefiltert wird. Die Umgebung muss daher durch einen darüber zu tragenden zusätzlichen MNS geschützt werden.

Ein Plexiglasgesichtsschild ist als mechanische Barriere in der Lage, Tröpfchen in beide Richtungen (vom Träger und von der anderen Person) aufzuhalten, bietet jedoch keinen Schutz gegen feine Aerosole. Es liegen keine Belege für eine Äquivalenz eines Visiers zu einer MNB vor [21]. Ein Vorteil ist ein zeitgleicher Schutz der Augen; in Einzelfällen wurde das SARS-CoV-2-Virus in Konjunktivalproben nachgewiesen [26]. Da SARS-CoV‑2 grundsätzlich über die Bindehaut und die Tränenkanäle übertragen werden kann, ist im Kontakt mit infizierten Personen das Tragen einer Schutzbrille insbesondere bei Aerosol-trächtigen Tätigkeiten erforderlich [25].

Bei allen Gesichtsbedeckungen muss auf den korrekten, eng anliegenden Sitz geachtet werden, um Leckagen zu vermeiden. Informationen zum korrekten Anlegen der persönlichen Schutzausrüstungen finden sich auf den Seiten des RKI und der Deutschen Gesetzlichen Unfallversicherung (DGUV). Spätestens nach Durchfeuchtung ist ein Wechsel der Gesichtsbedeckung erforderlich, da mit zunehmender Feuchte im Material die Filtereffizienz nachlässt [26]. Mund-Nasen-Bedeckungen sollten nach Benutzung vor Wiederverwendung bei mindestens 60 °C gewaschen werden. Medizinische Mund-Nasen-Schutze und Filtering-face-piece-Masken sind in der Regel Einmalprodukte und sollen nach Benutzung entsorgt werden [27]. Für den Fall, dass während einer Pandemie eine nicht ausreichende Anzahl von MNS oder FFP-Masken zur Verfügung steht, kann eine Wiederverwendung nach Trocknung und unter Beachtung von Hygienegrundsätzen erforderlich sein [24].

### Situationen, in denen das Tragen von Mund-Nasen-Bedeckungen und -Masken empfohlen wird

In Anlehnung an den SARS-CoV-2-Arbeitsschutzstandard des BMAS [2] wird das Tragen von Mund-Nasen-Bedeckungen bei nicht einhaltbaren Schutzabständen und einer darüber hinausgehenden persönlichen Schutzausrüstung in folgenden Fällen empfohlen:

- Im Kontakt mit nachweislich mit SARS-CoV-2-infizierten Personen ist Vollschutz (FFP2- oder FFP3-Maske, Schutzbrille, den Körper bedeckende Schutzkleidung) obligatorisch [28].
- Bei unvermeidbarem Kontakt zu Mitarbeiter*innen (< 1,5 m Abstand) tragen alle Beteiligten einen MNS. Bei eingeschränkter Verfügbarkeit des MNS kann alternativ eine Mund-Nasen-Bedeckung (MNB) zur Verfügung gestellt werden [2].
- Im Falle des unvermeidbaren, jedoch kontrollierbaren Kontakts zu einer fremden Person (<1,5 m Abstand) tragen Mitarbeiter*in und fremde Person einen MNS, wenn verfügbar. Bei Lieferengpässen von MNS soll alternativ zumindest eine Mund-Nasen-Bedeckung getragen werden.
- In Situationen mit unvermeidbarem, nichtkontrollierbarem Kontakt zu einer fremden Person (< 1,5 m Abstand), z. B. bei polizeilicher Festnahme oder Krankentransport einer verwirrten Person, soll der/die Mitarbeiter*in eine FFP2-Maske tragen, „Kund*innen“ tragen nach Möglichkeit MNS. Hierzu ist es erforderlich, dass entsprechende Berufsgruppen für den Bedarf eine FFP2-Maske bei sich tragen. Ein zusätzlicher Plexiglas-Gesichtsschutz kann vor Bespucken schützen.

## Umsetzung der betrieblichen Maßnahmen zum Infektionsschutz

Die Vermittlung der genannten Maßnahmen kann über öffentliche Empfehlungen, Publikationen oder Leitlinien erfolgen. Die Umsetzung in der Institution oder dem Betrieb ist durch die entsprechende Behördenleitung bzw. der Geschäftsführung sicherzustellen mit Beratung durch die Sicherheitsfachkraft, die Hygienefachkraft und die/den Betriebsärzt*in. Der/die Arbeitgeber*in kann seine/ihre Aufgaben, Pflichten und Verantwortlichkeiten im Arbeitsschutz an Führungskräfte oder andere Beauftragte delegieren. Entsprechende Unterweisungen der Mitarbeitenden sind zu empfehlen. Die den Maßnahmen zugrunde liegende Gefährdungsbeurteilung [3] kann mit Hilfe einer Muster-Gefährdungsbeurteilung [29] erstellt werden.

## Fazit für die Praxis

- Für systemrelevante Berufsgruppen des öffentlichen Dienstes sind persönlicher oder enger Kontakt mit der Bevölkerung und Kolleg*innen ggf. unvermeidbar. Um die Übertragung von SARS-CoV‑2 für Mitarbeitende und Risikogruppen einzugrenzen, sind spezifische Schutzmaßnahmen zur Erhöhung des Infektionsschutzes zu etablieren.
- In ausgewählten Fällen sind über die bevölkerungsbezogenen Basisempfehlungen (Abstandssicherung, Händehygiene, Husten- und Niesetikette) hinausreichende persönliche Schutzmaßnahmen erforderlich.
- Maßnahmen sollten dem STOP-Prinzip entsprechen: Vermeidung nicht notwendiger Kontakte, Einhalten der Hygiene- und Abstandsregeln, Reduzierung eventueller Quarantänefälle durch feste kleine Teams und der Situation angepasstes Tragen von persönlicher Schutzausrüstung.
- Definierte Ansprechpartner*innen und klare Kommunikation der betriebsintern vereinbarten Maßnahmen bei Auftreten eines Infektionsfalls unterstützen den Erhalt der Funktionsfähigkeit systemrelevanter Bereiche.

## Footnotes

1 *STOP*-Prinzip: *S*ubstitution, *t*echnische, *o*rganisatorische, *p*ersönliche (Schutz-)maßnahmen.

## References

[1] Bundesamt für Bevölkerungsschutz und Katastrophenhilfe (2009) Kritische Infrastruktur Definition und Übersicht. https://www.kritis.bund.de/SubSites/Kritis/DE/Einfuehrung/einfuehrung_node.html. Zugegriffen: 5. Nov. 2020

[2] Bundesministerium für Arbeit und Soziales (2020) SARS-CoV-2-Arbeitsschutzstandard. https://www.bmas.de/DE/Presse/Pressemitteilungen/2020/einheitlicher-arbeitsschutz-gegen-coronavirus.html. Zugegriffen: 16. Apr. 2020

[3] Bundesamt für Justiz (2019) Arbeitsschutzgesetz (ArbSchG), vom 7. August 1996 (BGBl. I S. 1246), zuletzt durch Artikel 113 des Gesetzes vom 20. November 2019 (BGBl. I S. 1626) geändert. https://www.gesetze-im-internet.de/arbschg/__5.html. Zugegriffen: 5. Nov. 2020

[4] Pieter J, Körner W, Harth V, Preisser AM (2020) Infektionsschutz im Öffentlichen Dienst. Kompetenznetz Public Health COVID-19. https://www.public-health-covid19.de/images/2020/Ergebnisse/2020_06_17_Handreichung__ffentlicher_Dienst_Kompetenznetz.pdf. Zugegriffen: 5. Nov. 2020

[5] Bundesministerium für Arbeit und Soziales (2020) Liste der systemrelevanten Bereiche. https://www.bmas.de/DE/Schwerpunkte/Informationen-Corona/Kurzarbeit/liste-systemrelevante-bereiche.html. Zugegriffen: 30. März 2020

[6] Bundesamt für Bevölkerungsschutz und Katastrophenhilfe (2020) Handlungsempfehlungen für Unternehmen, insbesondere für Betreiber Kritischer Infrastrukturen. https://www.kritis.bund.de/SharedDocs/Downloads/Kritis/DE/200302_HinweisePandemie.html. Zugegriffen: 6. Apr. 2020

[7] Robert-Koch-Institut (2020) Optionen zum Management von Kontaktpersonen unter Personal der kritischen Infrastruktur bei Personalmangel. https://www.rki.de/DE/Content/InfAZ/N/Neuartiges_Coronavirus/Personal_KritIs.html. Zugegriffen: 6. Nov. 2020

[8] Robert-Koch-Institut (2020) COVID-19-Verdacht: Maßnahmen und Testkriterien – Orientierungshilfe für Ärzte. https://www.rki.de/DE/Content/InfAZ/N/Neuartiges_Coronavirus/Massnahmen_Verdachtsfall_Infografik_Tab.html?nn=13490888. Zugegriffen: 6. Nov. 2020

[9] Occupational Safety and Health Administration (2020) Guidance on preparing workplace for COVID-19. OSHA 3990-03-2020. https://www.osha.gov/Publications/OSHA3990.pdf. Zugegriffen: 3. Juli 2020

[10] Dragano N, Hoffmann B et al (2020) Hintergrundpapier: Indirekte Gesundheitsfolgen der aktuellen Maßnahmen zum Infektionsschutz in Deutschland. https://www.public-health-covid19.de/images/2020/Ergebnisse/Hintergrundpapier_Indirekte_Folgen_von_Manahmen_des_Infektionsschutzes_Version01_23042020.pdf. Zugegriffen: 6. Nov. 2020

[11] Lengen J, Mache S (2020) Soziale Isolation im Home Office. https://www.public-health-covid19.de/images/2020/Ergebnisse/Handreichung_Soziale_Isolation_im_Homeoffice_2020_06_26.pdf. Zugegriffen: 6. Nov. 2020

[12] Riedel-Heller SG, Röhr S, Seidler A, Apfelbacher C (2020) Psychosoziale Folgen von Isolations- und Quarantänemaßnahmen: Womit müssen wir rechnen? Was können wir dagegen tun? https://www.public-health-covid19.de/images/2020/Ergebnisse/Policy_Brief_Psychosoziale_Folgen_von_Isolation_30042020_final.pdf. Zugegriffen: 6. Nov. 2020

[13] Robert-Koch-Institut (2020) Informationen und Hilfestellungen für Personen mit einem höheren Risiko für einen schweren COVID-19-Krankheitsverlauf. https://www.rki.de/DE/Content/InfAZ/N/Neuartiges_Coronavirus/Risikogruppen.html?nn=13490888. Zugegriffen: 29. Okt. 2020

[14] Angerer P, Kaifie-Pechmann A, Tautz A (2020) Beschäftigte mit erhöhtem Krankheitsrisiko. https://www.public-health-covid19.de/images/2020/Ergebnisse/Beschaftigte_mit_erhohtem_Krankheitsrisiko_Update_V2_AKPA_Neues_markiert_OK_PA_AK_LG_finale_Version-1.pdf. Zugegriffen: 6. Nov. 2020

[15] Seidler A, Petereit-Haack A (2020) Müssen ältere Beschäftigte dem Arbeitsplatz fernbleiben? https://www.public-health-covid19.de/images/2020/Ergebnisse/2020_04_23_Fact_Sheet_Auswirkungen_auf_ltere_Beschftigte_V3.pdf. Zugegriffen: 6. Nov. 2020

[16] Viswanathan M, Kahwati L, Jahn B, Giger K, Dobrescu A, Hill C, Klerings I, Meixner J, Persad E, Teufer B, Gartlehner G (2020). Universal screening for SARS-CoV-2 infection: a rapid review. Cochrane Database Syst Rev.

[17] Bundesamt für Justiz (2017) Biostoffverordnung (BioStoffV) vom 15. Juli 2013 (BGBl. I S. 2514), die zuletzt durch Artikel 146 des Gesetzes vom 29. März 2017 (BGBl. I S. 626) geändert worden ist, § 4 Gefährdungsbeurteilung. https://www.gesetze-im-internet.de/biostoffv_2013/__4.html. Zugegriffen: 5. Nov. 2020

[18] Robert-Koch-Institut (2020) Hinweise zu Reinigung und Desinfektion von Oberflächen außerhalb von Gesundheitseinrichtungen im Zusammenhang mit der COVID-19-Pandemie. https://www.rki.de/DE/Content/InfAZ/N/Neuartiges_Coronavirus/Reinigung_Desinfektion.html?nn=13490888. Zugegriffen: 3. Juli 2020

[19] Bundesindustrieverband Technische Gebäudeausrüstung (2020) Betrieb Raumlufttechnischer Anlagen unter den Randbedingungen der aktuellen Covid-19-Pandemie. 24.04.2020, Version 2. https://www.btga.de/files/Diverses/RLT_Covid19_V2_200424.pdf. Zugegriffen: 3. Juli 2020

[20] Robert-Koch-Institut (2020). Mund-Nasen-Bedeckung im öffentlichen Raum als weitere Komponente zur Reduktion der Übertragung von COVID-19. Strategie-Ergänzung zu empfohlenen Schutzmaßnahmen und Zielen. (3. Update). Epidemiol Bull.

[21] Robert-Koch-Institut (2020) Antworten auf häufig gestellte Fragen zum Coronavirus SARS-CoV-2/Krankheit COVID-19. Infektionsschutzmaßnahmen. https://www.rki.de/SharedDocs/FAQ/NCOV2019/gesamt.html?nn=13490888. Zugegriffen: 6. Nov. 2020

[22] Bundeszentrale für gesundheitliche Aufklärung (2020) Informationen rund um das Coronavirus. https://www.infektionsschutz.de/coronavirus.html. Zugegriffen: 6. Nov. 2020

[23] Deutsche Gesellschaft für Pneumologie und Beatmungsmedizin (2020) Stellungnahme der DGP zur Auswirkung von Nase-Mund-Masken auf den Eigen- und Fremdschutz bei aerogen übertragbaren Infektionen in der Bevölkerung. https://pneumologie.de/fileadmin/user_upload/COVID-19/2020-05-08_DGP_Masken.pdf. Zugegriffen: 5. Nov. 2020

[24] Bundesanstalt für Arbeitsschutz und Arbeitsmedizin (2014). Technische Regeln für Biologische Arbeitsstoffe – TRBA 250. Biologische Arbeitsstoffe im Gesundheitswesen und in der Wohlfahrtspflege.

[25] Bundesanstalt für Arbeitsschutz und Arbeitsmedizin (2020) Empfehlung organisatorischer Maßnahmen zum Arbeitsschutz im Zusammenhang mit dem Auftreten von SARS-CoV‑2, sowie zum ressourcenschonenden Einsatz von Schutzausrüstung. https://www.baua.de/DE/Themen/Arbeitsgestaltung-im-Betrieb/Coronavirus/pdf/Empfehlungen-organisatorische-Massnahmen.pdf?__blob=publicationFile&v=10. Zugegriffen: 5. Nov. 2020

[26] Koordinierungskreis für Biologische Arbeitsstoffe (KOBAS) der DGUV (2020) Empfehlung zur Tragezeitbegrenzung für Mund-Nase-Bedekcungen (MNB) im Sinne des SARS-CoV-2-Arbeitsschutzstandards un der SARS-CoV-2-Arbeitsschutzregel. Stellungnahme vom 27.05.2020, aktualisierte Fassung 07.10.2020. https://www.dguv.de/medien/inhalt/praevention/themen_a_z/biologisch/kobas/tragezeitbegrenzung_kobas_27_05_2020.pdf. Zugegriffen: 13. Nov. 2020

[27] Bundesanstalt für Arbeitsschutz und Arbeitsmedizin (2020) Empfehlungen der BAuA und des ad-Hoc AK „Covid-19“ des ABAS zum Einsatz von Schutzmasken im Zusammenhang mit SARS-CoV‑2. https://www.baua.de/DE/Themen/Arbeitsgestaltung-im-Betrieb/Coronavirus/pdf/Schutzmasken.pdf?__blob=publicationFile&v=17. Zugegriffen: 6. Nov. 2020

[28] Bundesanstalt für Arbeitsschutz und Arbeitsmedizin (2012) Beschluss 609: Arbeitsschutz beim Auftreten einer nicht ausreichend impfpräventablen humanen Influenza. https://www.baua.de/DE/Angebote/Rechtstexte-und-Technische-Regeln/Regelwerk/TRBA/pdf/Beschluss-609.pdf?__blob=publicationFile&v=2. Zugegriffen: 5. Nov. 2020

[29] Deutsche Gesetzliche Unfallversicherung, AGUM (2020) Muster-Gefährdungsbeurteilung nach Arbeitsschutzgesetz für den Schutz gegen die Ausbreitung von Krankheitserregern und die Aufrechterhaltung des Interimsbetriebs der Hochschulen. https://www.dguv.de/corona-bildung/hochschulen/muster-gefaehrdungsbeurteilung/index.jsp. Zugegriffen: 5. Nov. 2020 (gültig für die Feststellung einer epidemischen Lage von nationaler Tragweite (§5(1)IfSG):aktuell Coronavirus SARS-CoV-2)

